# Tortuous Ulnar Artery in the Distal Forearm

**DOI:** 10.7759/cureus.83522

**Published:** 2025-05-05

**Authors:** Shawhin R Shahriari, Cameron O'Brien, Kristopher Avant

**Affiliations:** 1 Department of Orthopedic Surgery, Integris Health, Oklahoma City, USA; 2 Orthopedic Surgery, College of Osteopathic Medicine, Oklahoma State University, Tulsa, USA; 3 Orthopedic Surgery, Oklahoma Center for Orthopedic and Multispecialty Surgery, Oklahoma City, USA

**Keywords:** forearm mass, hypothenar hammer, tortuous artery, tortuous ulnar artery, ulnar artery, variant ulnar artery, wrist mass

## Abstract

Vascular anomalies of the upper extremity are rare and often challenging to diagnose. The ulnar artery, a terminal branch of the brachial artery, typically traverses the forearm and enters the hand through Guyon's canal, where vascular pathologies have been implicated in ulnar tunnel syndrome. However, vascular anomalies occurring outside Guyon's canal, such as a tortuous ulnar artery in the distal forearm, are seldom reported and remain poorly understood. We report the case of a 57-year-old male with a palpable mass on the volar/ulnar aspect of his distal forearm, which was associated with intermittent pain during hand use. Surgical exploration of the suspected mass revealed a tortuous ulnar artery without evidence of vascular malformations. To alleviate symptoms and reduce palpability, the artery was buried beneath the fascia. Postoperatively, the patient experienced complete symptom resolution with no recurrence of the mass or symptoms at three months postoperatively. This case represents the third reported instance of a palpable, tortuous ulnar artery as a distal forearm mass. This highlights a rare clinical entity that resulted in symptomatic resolution with both diagnostic and therapeutic surgical intervention. This also exemplifies the need for a broad differential diagnosis, a step-wise approach, and intimate knowledge of anatomy when treating masses of the forearm.

## Introduction

Vascular anomalies of the upper extremity are rare and often challenging to diagnose. Typically, visual deformity or symptoms associated with the mass lead patients to seek care for such masses. Space-occupying lesions can cause compression neuropathy, which is distressing for the patient, and may lead to investigation and eventual diagnosis [1]. When evaluating masses of the distal forearm, physical exam and point-of-care ultrasonography are essential tools, along with reviewing the relevant anatomy.

The ulnar artery is a terminal branch of the brachial artery, which bifurcates at the proximal aspect of the forearm before coursing down the medial forearm. Distally, it enters the palm through the ulnar tunnel, also known as Guyon's canal, alongside the ulnar nerve and ulnar vein [2]. Guyon's canal is a fibro-osseous tunnel extending from the palmar carpal ligament at the proximal edge of the pisiform bone to the origin of the hypothenar muscles at the level of the hamulus [3].

The clinical significance of Guyon's canal is mainly linked to its potential to cause compression of the ulnar nerve while traveling through this canal. This commonly results in symptoms of pain and paresthesia in the distribution of the ulnar nerve, known as ulnar tunnel syndrome [2]. Space-occupying masses are a common cause of ulnar tunnel syndrome, with ganglion cysts being a leading cause [1]. Other causes of ulnar tunnel syndrome include hook of hamate fractures, anomalous hypothenar muscles, crystal deposition disease, and ulnar artery pathology [1]. A review of the current literature uncovered two cases of a tortuous ulnar artery causing compression of the ulnar nerve in Guyon's canal, leading to ulnar tunnel syndrome [2]. 

Hypothenar hammer syndrome is another related entity, a term coined in 1970, describing an aneurysm of the ulnar artery in the wrist, leading to symptoms including pulsatile mass in the hypothenar eminence, digital ischemia due to emboli, or asymptomatic ulnar artery occlusion [4]. Treatment for this entity ranges from conservative treatment and lifestyle modification to reconstruction with interpositional grafting of the occluded or aneurysmal segment [4]. Surgical intervention for this entity guides some of our thought process for reconstructing symptomatic aneurysms of the ulnar artery. 

In this report, we present a unique case of a palpable, tortuous ulnar artery in a healthy middle-aged male, highlighting its unique presentation, diagnostic workup, and the surgical management employed to address the patient's symptoms. This case underscores the importance of thorough evaluation and surgical exploration in rare vascular anomalies and offers insights into an effective technique for symptom relief.

## Case presentation

A 57-year-old right-hand-dominant male presented to the clinic with a mass on his right forearm on the volar/ulnar/distal aspect. At rest, his pain is a zero out of 10 on a visual analog pain scale. He noted increasing pain with the use of his right hand, and at worst, it is a five out of 10. He never had any surgery or recalled any specific trauma to the right upper extremity. Medical history is notable for medication-controlled hypertension.

On examination, the patient's right forearm mass appeared to be in the soft tissue. It did not transilluminate nor did it have a palpable thrill; however, there was a pulse that could be appreciated both on exam and using ultrasonography while in the clinic. The ultrasound demonstrated high flow through the mass; however, it was unclear if it was an aneurysm or pseudoaneurysm. Plain films of the wrist did not demonstrate any calcifications or phleboliths. We discussed surgical options with the patient, and he elected for exploration of the mass, with the possibility of vascular reconstruction of the ulnar artery if needed.

**Figure 1:**
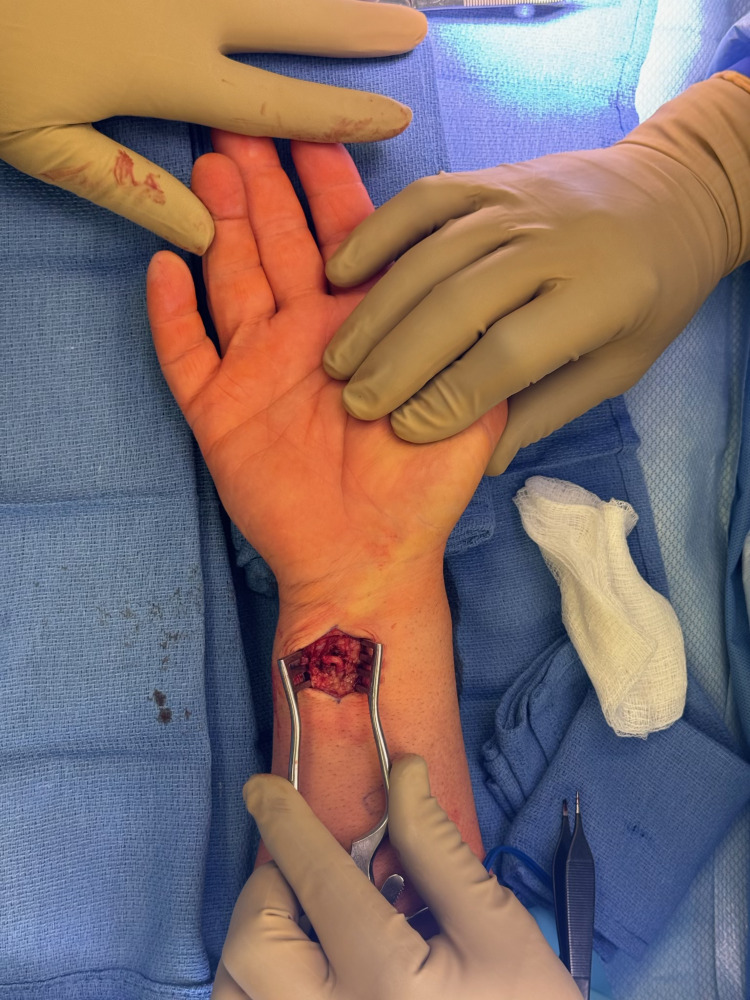
Intraoperative view of the tortuous ulnar artery including the hand for reference

**Figure 2:**
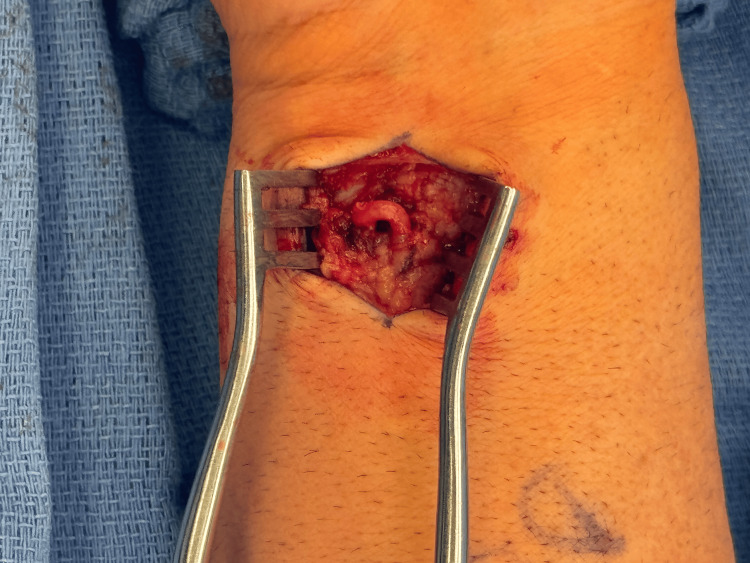
Close-up intraoperative view of the tortuous ulnar artery in the distal forearm

Intraoperatively, the skin was incised over the mass, and the ulnar artery was identified. The artery was dissected proximally and distally; however, no pseudoaneurysm or aneurysm was noted- the course of the ulnar artery was simply tortuous (Figures 1, 2). To reduce the palpability and to reduce the patient's symptoms, the artery was then buried under fascia, and the wound was then closed in layers.

Postoperatively, the patient's course went well, and he had sutures removed at two weeks. He did not note any further pain in his hand. He continues to demonstrate no recurrence of the mass or of any of his previous symptoms at three months post-operatively.

## Discussion

In terms of vascular lesions on the upper extremity, classically we describe aneurysm, pseudoaneurysm, and other vascular malformations; however, our patient's history was inconsistent with aneurysm and pseudoaneurysm as there was no previous trauma or cannulation of the ulnar artery, but they could not be completely ruled out. An option would have been to perform a magnetic resonance angiogram; however, this would have been time-consuming and would not have necessarily changed clinical decision-making. The ultrasound also demonstrated it was a high-flow lesion, and the plain films of the wrist demonstrated no phleboliths, thereby ruling out venous malformation [5].

We felt it was important to explore the mass for diagnostic and potentially therapeutic reasons. When we found it was not a typical vascular lesion, the decision was made to bury the ulnar artery with the goal of reducing the palpability of the artery; we were unsure if it would help with the patient's symptoms. Overly compressing the ulnar artery would have potentially caused further problems as well, so a vascular exam was performed at the end of the case to ensure adequate flow to the distal fingertips.

Other presentations of tortuous ulnar arteries include two that involve Guyon's canal [6,7]. Additionally, there is a description of two distal forearm masses (similar to our patient); however, these were in patients with significant chronic kidney disease [8]. These reports found that the ulnar artery did not have any aneurysm or pseudoaneurysm, but rather had a tortuous appearance. There is no clear answer to the surgical management of these, and most of these arteries were simply left alone. We found raising the local muscle fascia allowed for mobilization of the artery, allowing it to be buried and for the patient to no longer feel the mass so superficially.

## Conclusions

In this study, we discuss an unusual presentation of a palpable, tortuous ulnar artery causing intermittent pain in the distal forearm. To our knowledge, this is the third time this entity has been reported in the literature, and the first time it has been described as symptomatic while also outside of Guyon's canal. This rare diagnosis can be difficult to identify, and surgical exploration of these masses can be both diagnostic and therapeutic. We found burying the artery in the local muscle fascia to be an effective technique for reducing pain and palpability of the tortuous ulnar artery. We would advocate for a hand surgeon to evaluate and treat these patients, with the requisite knowledge of the local anatomy and the existence of such entities in order to treat this type of presentation appropriately.
